# Variations in Pedicel Structural Properties Among Four Pear Species (*Pyrus*): Insights Into the Relationship Between the Fruit Characteristics and the Pedicel Structure

**DOI:** 10.3389/fpls.2022.815283

**Published:** 2022-01-31

**Authors:** Zhenhua Cui, Haoqi Sun, Yuqin Lu, Lixin Ren, Xinrui Xu, Dingli Li, Ran Wang, Chunhui Ma

**Affiliations:** ^1^College of Horticulture, Qingdao Agricultural University, Qingdao, China; ^2^Engineering Laboratory of Genetic Improvement of Horticultural Crops of Shandong Province, Qingdao, China; ^3^Sanying Precision Instruments Co., Ltd, Tianjin, China

**Keywords:** fruit characteristics, pedicel structure, *Pyrus*, 3D observation, vessels

## Abstract

Fruit pedicel is the bridge linking the parent tree and the fruit, which is an important channel for water and nutrients transport to the fruit. The genetic specificity determines the characteristics of the pedicel and the fruit, but the relationship between the pedicel structure and the fruit characteristics is unexplored. Combining the investigation of fruit characteristics, the statistical analysis of the pedicel structural properties, and the 2D and 3D anatomical observation of the pedicel, this study found distinctive contributions of the pedicel elements to the fruit characteristics in four pear species. The European pear (Conference) showed distinct fruit shape index and pedicel structural properties compared with the oriental pears (Akizuki, Yali, and Nanguoli). The fruit size positively correlated with pedicel length, fiber area, pedicel diameter, the area percentage of the cortex, and the area percentage of phloem; however, fruit firmness and soluble solids concentration are showed a stronger positive correlation with xylem area, pith area, the area percentage of xylem, the area percentage of sieve tube, and the area percentage of pith. Pedicel elements, including pith, fiber, and cortex, likely play a certain role in the fruit growth due to the variations of their characteristics demonstrated in the four pear species. The porosity, the ratio of the surface area to the volume, and the spatial arrangement of the vessels showed significant variations across the pear species, indicating the distinction of the hydraulic conductance of the pedicels. Our findings provided direct evidence that pedicel structural elements contributed distinctively to the fruit characteristics among pear species.

## Introduction

In fruit trees, the pedicel is a bridge linking the parent tree and the fruit, through which the water, mineral nutrients, and photosynthate are transported to the fruit. Therefore, the fruit characteristics are highly affected by the efficiency of water and nutrient transport *via* the pedicel, which is determined by the structural properties of the pedicel. In the early stage of the fruit growth, water enters the fruit predominantly *via* the xylem (Greenspan et al., 1994, 1996) and thereafter mainly through the phloem as the xylem function slows down gradually along with the development of the fruit (Bondada et al., 2005). Mineral elements present in soil enter the root apoplast path and are transported along with the mass flow of water to the fruit through the xylem pathway (Gilliham et al., 2011), while the photosynthate is delivered to the fruit *via* the phloem pathway. Therefore, the structural properties of both xylem and phloem of the pedicel are important in affecting the fruit growth.

In a study with nine fruit species, vessel density, size, and area in the pedicel showed quite variations among them, but the loss of xylem functionality along with the fruit maturity and higher Ca content in the pedicel than the fruit were found as universal patterns, indicating the existence of a pedicel-fruit “bottleneck” effect in Ca transport across species (Song W. et al., 2018). In another study of fruit pedicel, the Ca content in the phloem was even higher than that in the xylem during the fruit growth, such as litchi, indicative of possible Ca delivery to fruit also *via* phloem mass flow (Song W. P. et al., 2018). Other lines of evidence demonstrated the correlation between pedicel diameter and fruit size at harvest (Bustan et al., 1995; Nii, 1998), and it has been proposed that the pear fruit adjusted the development of the vascular bundle of the pedicel as the fruit grew from the young to mature stage (Nii, 1980). The cotton growth was impaired by the decrease of carbohydrate translocation due to the alteration of the pedicel structure (de Oliveira et al., 2006). In grape, hydraulic conductance between the berry and pedicel declined substantially at later ripening stages predominantly due to the decline in pedicel conductance (Choat et al., 2009; Knipfer et al., 2015). In tomatoes, the hydraulic properties of the pedicel showed significant developmental changes, which was believed to be associated with the anatomical changes of xylem (Lee, 1989; Van Ieperen et al., 2003; Rančić et al., 2008, 2010). All these observations indicate that the fruit quality is highly associated with the transport capacity of the pedicel, which is determined by its structural properties, especially of xylem and phloem. However, the influence of the transport capacity of the pedicel on the fruit growth is still a matter of controversy. For example, Zhang et al. (2005) reported that photosynthate accumulation during the period of the rapid fruit growth is limited by the sink strength of fruit rather than by the transport capacity of the pedicel in pear. Even though it is known that different cultivars present great variations in fruit appearances, flavors, nutrient substances, and maturity period, as well as morphological characteristics of their pedicel due to the genetic control, the relationship between the pedicel structural properties and fruit traits is still not fully understood and needs further exploration.

Pear (*Pyrus* spp.) is a commercially important and worldwide popular fruit crop. *Pyrus communis*, *Pyrus breschneideri*, *Pyrus pyrifolia*, and *Pyrus ussuriensis* are the four main cultivated species in pear-producing regions worldwide. Both the fruit characteristics and the pedicel morphological features of the four species show significant variations. Combining the investigation of fruit characteristics, microscopic observation and hydraulic conductance measurement of the pedicel, and the X-ray computed microtomography analysis of the pedicel structure, this study was conducted to obtain a comprehensive understanding of the relationship between the pedicel structural properties and the fruit characteristics.

## Materials and Methods

Four 10-year-old pear species, namely, Conference (*P. communis*), Akizuki (*P. pyrifolia*), Yali (*P. breschneideri*), and Nanguoli (*P. ussuriensis*), were used in this study. The trees were planted with 2 m space within rows and 5 m space between rows with the normal fertilization and irrigation management as the commercial orchard does at Jiaozhou Experimental Station of Qingdao Agricultural University, located at 36°19′N and 120°23′E in Shandong Province, China. Trees were pruned every December with a delayed-open central leader system. The soil pH was about 6.5. Five trees of each species were used for fruit collection.

### Analysis of Fruit Characteristics

Thirty fruits of each cultivar were collected at harvest for the characteristics analysis. To obtain a unified biological maturity, the fruit of Conference, Akizuki, Yali, and Nanguoli were harvested at 120, 135, 150, and 130 days after full bloom, respectively. Fruit size was determined by measuring the vertical and transverse diameters using an electronic caliper (Mitutoyo, Japan). Fruit shape index was presented as the ratio of vertical diameter to transverse diameter. Fruit firmness and soluble solids concentration were measured according to Cui et al. (2020).

### Analysis of Pedicel Anatomy

Pedicels were collected from mature fruits for the structural analysis. The length and diameter of the pedicel were measured using an electronic caliper (Mitutoyo, Japan). The middle portion of the fresh pedicel was used for frozen sectioning according to Kawamoto (2003) with some modifications. In brief, fresh pedicels were fixed in 70% ethanol for 2 h and then transferred to 5% glycerol for 2 h followed by rapid freezing in the precooled container at –15°C. An optimal cutting temperature (OCT) compound (No. 4583, Sakura, United States) was used as an embedding medium. The frozen OCT compound block was then fixed on the sample stage for sectioning using a cryostat (CM1950, Leica, Germany). The section thickness was set at 10 μm. The sections were stretched on the glass slide at room temperature for 10 min. Then, the sections were stained with 5 mg/ml trypan blue (dissolved in 2% acetic acid) for 3 min, followed by 10 mg/ml acridine red (dissolved in 50% ethanol) for 5 min and 10 mg/ml acridine yellow (dissolved in 2% acetic acid) for 40 s. After rinsing with distilled water for 2 min, the tissues were covered with 1 ml of 50% glycerol before being observed under the fluorescent microscope (DM2500, Leica, Germany). Different tissues from the transverse sections were recognized, and their areas were calculated using the Leica Application SuiteX 3.4.2 software (Leica, Germany).

### X-Ray Computed Tomography Scanning

To visualize the microstructure of the pedicels three-dimensionally (3D) and nondestructively at high resolution, the pedicels were imaged using the nanoVoxel-3502E system (Sanying Precision Instruments Co., Ltd., Tianjin) after being collected from the fruit. The scanning was conducted with the parameters as shown in Table 1. Transverse and longitudinal section slices were generated from the shadow projections using the Feldkamp reconstruction algorithm (Feldkamp et al., 1984). The pedicel was rotated on the stage at an increment of 0.2°C over a total of 360°C at room temperature, yielding 1,800 2D projection images. The 2D projection images were reconstructed using the OCTOPUS 8.6 software (Institute for Nuclear Sciences, Ghent University, Ghent, Belgium), and the reconstructed 3D images were visualized using the Avizo 8.1 software (Thermo Fisher Scientific, China).

**TABLE 1:** Settings of X-ray scanning used in this study.

Sample	SOD/SDD (mm/mm)	Pixel size (μm)	Voltage (kV)	Current (μA)	Scanning time (h)
Conference	3.83/148.15	1.30	60	40	0.3
Akizuki	2.63/137.88	0.96	60	35	0.3
Yali	4.83/164.97	1.47	60	70	0.3
Nanguoli	3.83/164.01	1.18	60	70	0.3

*SOD, source origin distance; ODD, source origin detector distance.*

To differentiate the structural properties of the four pear species, the scanning data were extracted from a 100 μm × 100 μm × 300 μm volume of the pith, vessel, fiber, and cortex tissues, respectively, for the surface area and porosity analysis using the volume-rendering module function of the Avizo 8.1 software. The surface area of the analyzed tissue included the exterior surface and the interior surface. The surface area was calculated as the number of pixels on the surface multiplied by the pixel size. To recognize the pore space, the global threshold of the grayscale limit (5,840) was used as a discriminative value for the separation of the pores (grayscale value < 5,840) and the solid tissues (grayscale value > 5,840). Porosity was defined as the pore volume/area divided by the total volume/area of the analyzed sample.

For a more detailed analysis of the structural and spatial arrangement of the vessels, a combination of virtual longitudinal sections and 3D renderings was applied to visualize the intervessel connections. The 3D network of the vessels was skeletonized using the Avizo 8.1 software to extract the essential connectivity information between vessels.

### Analysis of Pedicel Hydraulic Conductance

The pedicel hydraulic conductance was analyzed according to the Darcy’s law as described in Brüggenwirth and Knoche (2015):


$$K_{\text{h}}=\frac{\Delta \,\text{V}}{\Delta \,\text{P}\cdot \Delta \,\text{t}}$$

where K_h_ is hydraulic conductance in m/s/MPa. To measure the value of other parameters, a simple device composed of a pump, a pressure transducer, an adapter, and two silicon tubes was used to produce a constant pressure (about 0.3 MPa, ΔP) by pumping 0.1 M CaCl_2_ solution on one side of the pedicel. CaCl_2_ solution pushed through the pedicel was collected on the other side, and the time (Δt) and volume of CaCl_2_ (ΔV) through the pedicel were recorded.

### Data Analysis

The principal component analysis (PCA) was conducted based on the data of fruit characteristics and the structural properties of the pedicel using the R (4.1.0) software. The group correlation analysis between fruit characteristics and pedicel structural properties was conducted using the R (4.1.0) software. The ANOVA and significant comparison were performed using the DPS 7.05 software (Zhejiang University) using the Tukey’s multiple range test.

## Results

### Comparison of Fruit Characteristics

**FIGURE 1:**
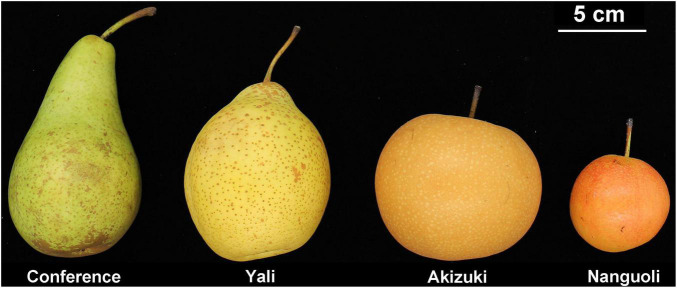
The morphological appearance of the four pear species used in this study at maturity. From left to the right are *P. communis*, *P. breschneideri*, *P. pyrifolia*, and *P. ussuriensis*.

Among the four pear species, Akizuki had the greatest single fruit weight (344 g), followed by Conference (251 g), Yali (223 g), and Nanguoli (102 g) with significant differences between each of them (Table 2). The vertical diameter comparison of the fruit was Conference (118 mm) > Yali (84 mm) > Akizuki (70 mm) > Nanguoli (54 mm), and Akizuki has the greatest transverse diameter (85 mm), followed by Yali (73 mm), Conference (68 mm), and Nanguoli (58 mm) with significant difference between each of them (Figure 1); accordingly, the fruit shape index value from high to low was Conference (1.783), Yali (1.14), Nanguoli (0.94), and Akizuki (0.83). Nanguoli had the greatest fruit firmness, followed by Yali (Figure 1); Conference and Akizuki had a similar level of fruit firmness, both of which were lower than Yali. Nanguoli had the highest soluble solids concentration (14.88%), followed by Conference (13.21%), Akizuki (12.62%), and Yali (11.78%) (Table 2).

**TABLE 2:** Fruit morphological and quality characteristics of the four pear species.

Cultivars	Single fruit weight (g)	Vertical diameter (mm)	Transverse diameter (mm)	Fruit shape index	Fruit firmness (kg/cm^2^)	Soluble solids concentration (%)
Conference (*P. communis*)	251.23 ± 36.13 b	118.95 ± 6.93 a	68.72 ± 3.78 c	1.73 ± 0.08 a	5.68 ± 2.00 c	13.21 ± 0.69 b
Akizuki (*P. pyrifolia*)	334.44 ± 12.78 a	70.77 ± 3.89 c	85.34 ± 2.94 a	0.83 ± 0.07 d	5.74 ± 1.02 c	12.62 ± 1.57 bc
Yali (*P. bretschneideri*)	223.10 ± 27.71 c	84.15 ± 6.94 b	73.65 ± 5.48 b	1.14 ± 0.06 b	8.13 ± 1.67 b	11.78 ± 0.85 c
Nanguoli (*P. ussuriensis*)	102.61 ± 9.73 d	54.53 ± 3.11 d	58.19 ± 2.36 d	0.94 ± 0.04 c	13.1 ± 1.09 a	14.88 ± 0.60 a

*Means within a column followed by different letters are significantly different at p < 0.05 using the Tukey’s multiple range test.*

### Analysis of Pedicel Anatomy

**FIGURE 2:**
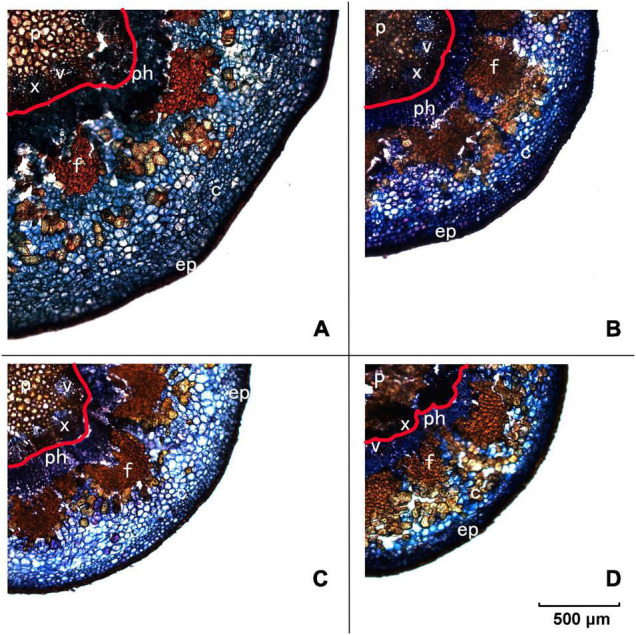
Frozen transverse sections of pedicels observed under the fluorescent microscope. Panel **(A)** is Conference, panel **(B)** is Yali, panel **(C)** is Akizuki, and panel **(D)** is Nanguoli, respectively. p, pith; x, xylem; v, vessels; ph, phloem; f, fiber cap; c, cortex; ep, epidermis; the area outside the red line on each panel was designated as the phloem area for the calculation of the area ratio.

To give a detailed analysis of the structural property of the pedicel, frozen sectioning was conducted (Figure 2). As observed in Figure 1, Yali had the greatest length of the pedicel (43 mm), followed by Conference (35 mm), Akizuki (28 mm), and Nanguoli (23 mm) (Table 3). Conference showed a greater diameter (3.66 mm) than that of Akizuki (3.27 mm) and Yali (3.27 mm), while the diameter of Nanguoli (2.31 mm) was the smallest (Table 3 and Figure 2); accordingly, Conference had a significantly greater transverse area of the pedicel (9.94 mm^2^), followed by Akizuki (6.62 mm^2^), Yali (6.43 mm^2^), and Nanguoli (5.23 mm^2^) (Figure 2 and Table 3). The pith, xylem, vessels, phloem, fiber, cortex, and epidermis tissues were marked out (Figure 2), and their area proportion relative to the pedicle transverse area was calculated as shown in Table 3; Conference had a significantly higher ratio of phloem area than Nanguoli (Table 3), but the xylem area ratio of Conference was lower than Nanguoli; Akizuki had a higher ratio of vessel area than Conference but had no difference with Yali and Nanguoli; compared with Akizuki and Nanguoli, Conference had a higher ratio of cortex area; Nanguoli had higher ratios of pith and sieve tube area; the ratio of fiber area in Akizuki was higher than that of Conference (Table 3).

**TABLE 3:** Pedicel morphological and anatomical parameters of the four pear species.

Pedicel structural properties	Conference (*P. communis*)	Akizuki (*P. pyrifolia*)	Yali (*P. bretschneideri*)	Nanguoli (*P. ussuriensis*)
Pedicel length (mm)	35.30 ± 3.49 b	28.33 ± 6.28 c	43.43 ± 3.72 a	23.62 ± 4.06 d
Pedicel diameter (mm)	3.66 ± 0.29 a	3.27 ± 0.31 b	3.27 ± 0.24 b	2.31 ± 0.16 c
Pedicel length/pedicel diameter	11.15 ± 1.24 b	5.75 ± 1.75 c	16.29 ± 2.78 a	10.33 ± 2.32 b
Pedicel area (mm^2^)	9.94 ± 1.82 a	6.62 ± 0.43 b	6.43 ± 0.33 b	5.23 ± 0.85 b
Xylem area (mm^2^)	0.82 ± 0.12 a	0.73 ± 0.11 a	0.76 ± 0.03 a	0.88 ± 0.42 a
Phloem area (mm^2)^	9.12 ± 1.70 a	5.89 ± 0.51 b	5.68 ± 0.31 b	4.34 ± 0.43 b
Pith area (mm^2^)	0.39 ± 0.17 a	0.23 ± 0.06 a	0.33 ± 0.06 a	0.53 ± 0.31 a
Vessel area (mm^2^)	0.43 ± 0.09 a	0.50 ± 0.05 a	0.43 ± 0.02 a	0.35 ± 0.11 a
Sieve tube area (mm^2^)	0.93 ± 0.17 a	0.78 ± 0.12 ab	0.64 ± 0.01 b	0.70 ± 0.02 ab
Fiber area (mm^2^)	1.16 ± 0.17 ab	1.30 ± 0.06 a	1.03 ± 0.10 bc	0.87 ± 0.07 c
Cortex area (mm^2^)	6.49 ± 0.95 a	3.81 ± 0.50 b	4.01 ± 0.20 b	2.78 ± 0.34 b
Xylem%	8.25 ± 0.22 b	11.16 ± 1.3 ab	11.80 ± 0.46 ab	16.47 ± 2.19 a
Phloem%	91.74 ± 0.38 a	88.83 ± 2.28 ab	88.20 ± 0.46 ab	83.52 ± 3.80 b
Pith%	3.83 ± 0.81 b	3.58 ± 0.70 b	5.07 ± 0.43 ab	9.79 ± 1.76 a
Vessel%	4.42 ± 0.80 b	7.56 ± 0.62 a	6.73 ± 0.33 ab	6.68 ± 0.43 ab
Sieve tube%	9.34 ± 0.15 b	11.73 ± 0.70 ab	9.94 ± 0.23 b	13.52 ± 0.75 a
Fiber%	11.97 ± 1.55 b	19.79 ± 1.2 a	15.98 ± 0.52 ab	16.68 ± 0.59 ab
Cortex%	65.60 ± 2.15 a	57.31 ± 2.60 bc	62.27 ± 0.06 ab	53.25 ± 0.84 c

*Means within a column followed by different letters are significantly different at p < 0.05 using the Tukey’s multiple range test.*

### Correlation Analysis Between Fruit Quality and Pedicel Property

**FIGURE 3:**
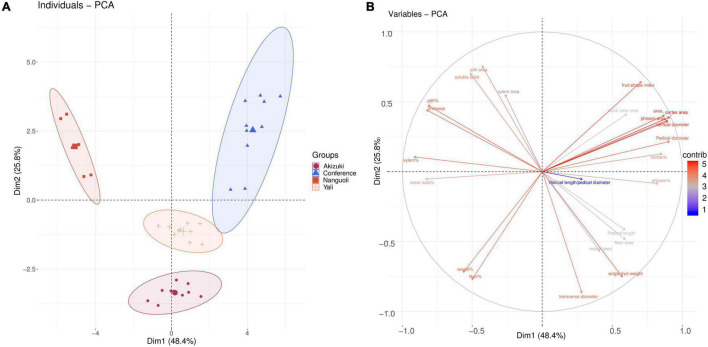
Clustering analysis of the pear species. **(A)** The principal component analysis (PCA) score of the four pear species based on their fruit characteristics and pedicel structural properties parameters; **(B)** loading plot of **(A)** according to the contribution of the variables in panel **(A)** model.

The PCA was conducted to see the clustering of the pear species (Figure 3) based on the fruit characteristics (Table 1) and structure data of the pedicel (Table 3). The PCA scoring plot showed apparent evidence of sample grouping with a 74.2% explanation of the total variance by PC1 (48.4%) and PC2 (25.8%), respectively, (Figure 3A). The loading plot indicated that the cortex area, phloem area, pedicel area, fruit shape index, and fruit vertical diameter are the top five contributing factors (Figure 3B) in the PCA model (Figure 3A).

**FIGURE 4:**
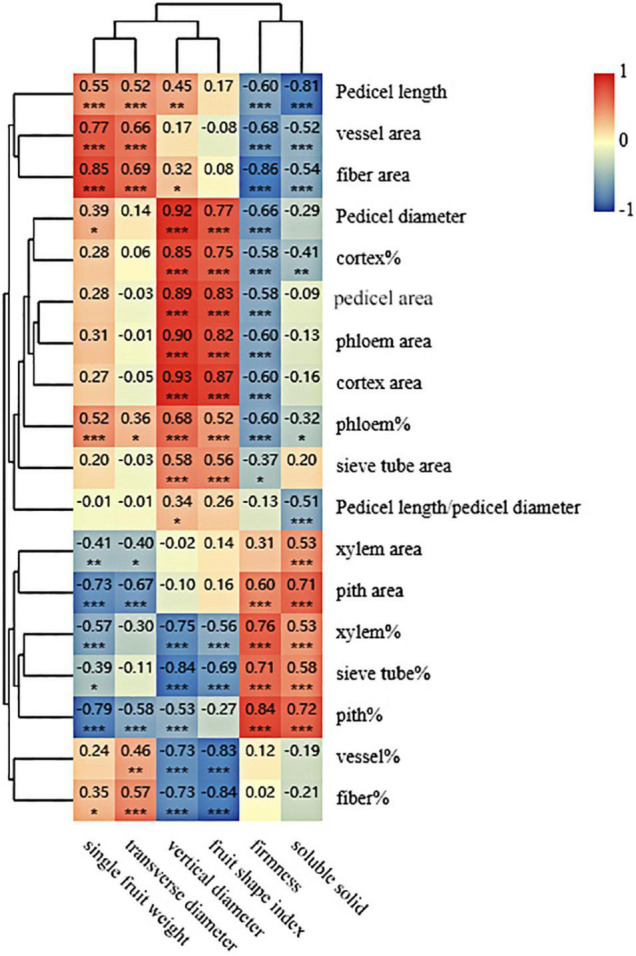
Clustering and correlation analysis between the fruit characteristics data and the pedicel structural parameters. The figures shown are Pearson’s correlation coefficients at 2 tails. *, **, and *** mean correlation is significant at 0.5, 0.01, and 0.001 level, respectively.

The correlation analysis between two groups (i.e., fruit characteristics and pedicel structural parameters) was completed (Figure 4). As shown in Figure 4, a strong correlation was found between fruit characteristics and structural parameters of the pedicel. Fruit soluble solids concentration had a positive correlation with xylem area, pith area, the area percentage of xylem (xylem%), the area percentage of sieve tube (sieve tube%), and the area percentage of pith (pith%); however, pedicel length, vessel area, fiber area, the area percentage of cortex (cortex%), the area percentage of phloem (phloem%), and pedicel length/pedicel diameter showed a negative correlation with soluble solid concentration of fruit. Compared with fruit soluble solids concentration, fruit firmness had a similar correlation pattern to the structural parameters of the pedicel. For the fruit shape index and vertical diameter, both of them had a positive correlation with pedicel diameter, cortex%, pedicel area, phloem area, cortex area, phloem%, and sieve tube area, and they both showed a negative correlation with xylem%, sieve tube%, the area percentage of the vessel (vessel%), and the area percentage of fiber (fiber%). Fruit transverse diameter and single fruit weight had a similar correlation with the pedicel structural parameters, both of which had a positive correlation with pedicel length, vessel area, fiber area, phloem%, and fiber% and negative correlation with xylem area, pith area, and pith% (Figure 4).

### X-Ray CT Scanning

**FIGURE 5:**
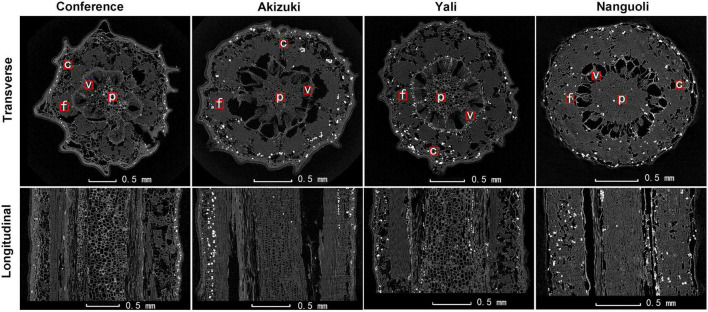
Representative transverse and longitudinal X-ray CT images of the fresh pedicel. Black areas indicate the air-filled tissue, dark gray areas indicate plant tissue, and white spots indicate water-filled tissue. f, fiber cap; p, pith; v, vessels; c, cortex.

To see details of the pedicel structural property, both the transverse and longitudinal 2D projections were extracted as shown in Figure 5. From all the transverse 2D projections, tissues of pith, vessel, fiber, and cortex were recognized as indicated in the observation of the frozen section (Figure 2). The area ratios of the different tissues varied apparently among the four species (Figure 5), which were consistent with the results shown in Table 3. In both transverse and longitudinal projections, Conference and Yali showed a more porous pith structure than Akizuki and Nanguoli (Figure 5).

**FIGURE 6:**
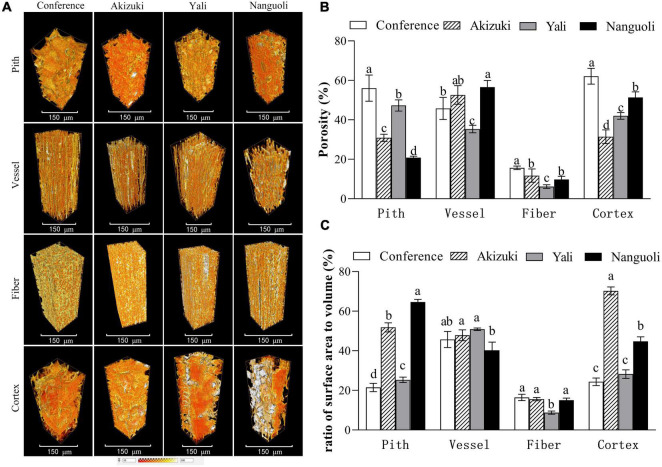
Structural analysis of the pith, vessel, fiber, and cortex of the pedicels. **(A)** The 3D renderings were reconstructed from 100 μm × 100 μm × 300 μm volume of each tissue; **(B)** porosity analysis of the different tissues as shown in panel **(A)**; **(C)** analysis of the surface area of the tissues as shown in panel **(A)**; the ratio of the surface area to the volume was calculated as the value of the surface area divided by the volume of the analyzed tissue. Bars within a group with different letters on the top are significantly different at *p* < 0.05 using the Tukey’s multiple range test.

Representative 3D images showed volume renderings of the pith, vessel, fiber, and cortex tissues from a 100 μm × 100 μm × 300 μm area (Figure 6A). 3D renderings displayed different morphological characteristics of the tissues. Fiber tissues showed a more regular and compact shape, followed by vessel tissues; however, the pith and cortex tissues presented irregular and uneven shapes of 3D renderings (Figure 6A). For a porosity analysis, there were interspecific differences in different tissues. Conference had the greatest porosity in the pith, followed by Yali, Akizuki, and Nanguoli (Figure 6B); Nanguoli had a greater porosity in the vessel than Conference and Yali (Figure 6B); Conference had a greater porosity in fiber than Akizuki and Nanguoli, and Yali had the smallest porosity in fiber (Figure 6B); Conference had the greatest porosity in the cortex, followed by Nanguoli, Yali, and Akizuki (Figure 6B). For the ratio of surface area to volume, Nanguoli had a greater ratio of surface area to volume in the pith, followed by Akizuki, Yali, and Conference (Figure 6C); Fiber tissue had a remarkably lower ratio than the pith, vessel, and cortex (Figure 6C), which was consistent with the low porosity in fiber compared with other three tissues (Figure 6B); and Yali had a lower ratio of surface area to volume in fiber tissue than other three species (Figure 6C); Conference, Akizuki, and Yali had similar ratios of surface area to volume in the vessel (Figure 6C); Akizuki had the greatest ratio of surface area to volume in the cortex, followed by Nanguoli, Conference, and Yali (Figure 6C).

**FIGURE 7:**
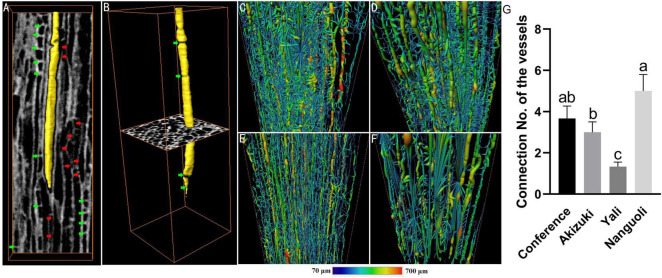
Structural and spatial arrangement of xylem vessels in the pedicel by X-ray scanning. **(A)** Longitudinal slice of the vessel tissue, light gray areas indicate the vessel walls, dark gray areas indicate the vessel lumens, one of which was rendered in yellow; red arrows indicate the location of intervessel connection; green arrows indicate the interruptions of the vessel lumen; **(B)** a representative 3D-reconstructed vessel isolated from surrounding vessels; panels **(C–F)** are the 3D-reconstructed skeleton of the vessels of Conference, Akizuki, Yali, and Nanguoli, respectively; **(G)** intervessel connectivity analysis, connection no. means the average no. of intervessel connections. Bars with different letters on the top are significantly different at *p* < 0.05 using the Tukey’s multiple range test.

The intervessel connection and network information of the vessels were analyzed by high-resolution observation, 3D image reconstruction, and skeletonization (Figure 7). From the view of a representative 2D projection, vessel elements displayed an irregular arrangement pattern with different shapes, length, width, angles, and thickness of the vessel wall (Figure 7A); as indicated by green arrows, interruptions of the vessel lumen were observed (Figure 7A); intervessel connections, as indicated by red arrows, can be detected by X-ray CT scanning, which extended the pathway of the vessels (Figure 7A); a solitary vessel isolated from surrounding vessels was rendered in a 3D image, which also indicated the irregular shape of the vessel and the interruption inside the vessel lumen (Figure 7B); skeletonization demonstrated the 3D network model of the vessels in four species (Figures 7C–F), indicating different levels of connectivity of the vessels; Nanguoli showed a highly branched and more connected network of vessels; for a more detailed analysis, Nanguoli showed the highest level of connectivity with 5 connected locations on each vessel, followed by Conference, Akizuki, and Yali (Figure 7G).

### Pedicel Hydraulic Conductance

**FIGURE 8:**
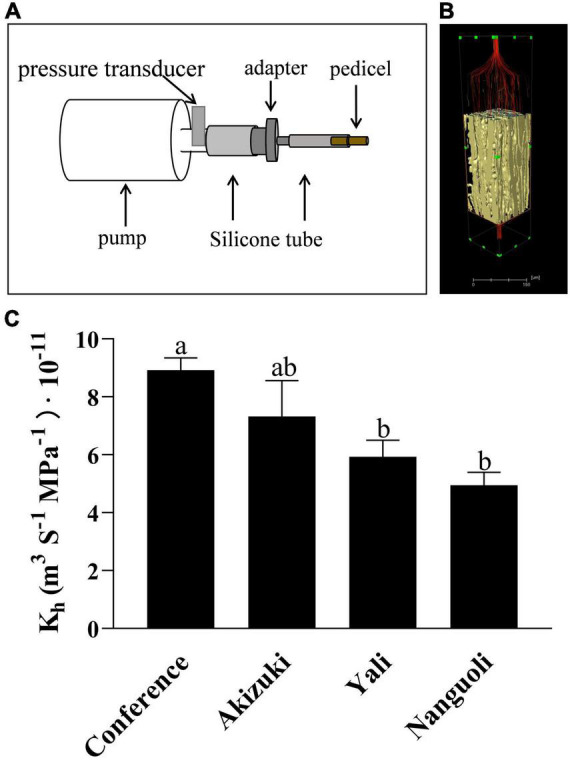
Hydraulic conductance analysis of the pedicel. **(A)** Schematic diagram of the device used to estimate the hydraulic conductance in pear pedicels. CaCl_2_ solution was pushed by the pump through the pedicel from left to the right side, the time and the CaCl_2_ volume passing through the pedicel were recorded; **(B)** a virtual model of the pedicel, where the flow behavior is laminar, and Darcy’s law is valid for the hydraulic conductance analysis; **(C)** hydraulic conductance of different pear pedicels according to Darcy’s law. Bars with different letters on the top are significantly different at *p* < 0.05 using the Tukey’s multiple range test.

As shown in Figure 8, the pedicel hydraulic conductance was estimated using a simple device (Figure 8A) according to Darcy’s law (Figure 8B). For the four pear species, Conference had the highest hydraulic conductance in the pedicel, followed by Akizuki (Figure 8C); Yali and Nanguoli had similar levels of hydraulic conductance of the pedicels, both of which were lower than that of Conference (Figure 8C).

## Discussion

Various anatomical techniques have been applied to study the structural properties of pedicel including grape (Knipfer et al., 2015), apple (Drazeta et al., 2004; Horbens et al., 2015), tomato (Rančić et al., 2010; Li et al., 2021), litchi (Song W. et al., 2018; Song W. P. et al., 2018), and pepper (Setiamihardja and Knavel, 1990), indicative of the importance of pedicels to the fruit development and growth. However, there have been limited studies combing various anatomical techniques to study the function of the pedicel (Rančić et al., 2010). Based on the 2D histological and 3D observation, this study analyzed the interspecific variations of structural properties of the pear pedicel, which also provided novel insights into the relationship between the structural property of the pedicel and the fruit characteristics.

Interestingly, the Conference showed a distinct fruit shape index (Figure 1 and Table 2) and pedicel structural properties (i.e., distribution and density of the vessels) (Figure 2 and Figure 5) compared with the other three species in our study, implying the genetic specificity between the European and the oriental pears. This was also supported by the PCA results (Figure 3) based on the fruit characteristics and structural properties data of the pedicel. As a terminal organ, fruit acquires nutrients and photosynthetic assimilates to support the enlargement and quality formation. For this process, the fruit pedicel behaved as a pivotal channel linking the parent body (i.e., source) and the fruit (i.e., sink). Differences in both fruit characteristics and pedicel structural properties were obvious among the investigated pear species in our study (Figures 1, 3 and Tables 1, 2). Fruit vertical diameter is the determinant of the fruit shape index, which showed a highly positive correlation with pedicel length, fiber area, pedicel diameter, cortex%, and phloem%. This interesting finding is hard to explain since both water and nutrients are delivered to the fruit through the pedicel. Water uptake accounts more for the fruit expansion; therefore, those above parameters (i.e., pedicel length, fiber area, pedicel diameter, cortex%, and phloem%) possibly affected water uptake into the fruit profoundly. Bussières (2002) found water import in young tomato fruit is limited by the low potential of the water entering the fruit due to pedicel resistance and calyx transpiration. But it is not sure if the variations of those parameters affected the pedicel resistance and calyx transpiration in our study. To the best of our knowledge, few studies focusing on the correlation between pedicel structural properties and fruit characteristics have been reported. In oriental pear Wonhwang, quick enlargement of xylem and phloem tissues in pedicel by GA treatment during the fruit development resulted in the increased size of the fruit (Park and Park, 2017), which was consistent with the results in our study, and this process was primarily due to the increment of cell division of both xylem and phloem. In tomato, Li et al. (2021) found that the water deficit did not change the hydraulic resistance and the vascular structure in the pedicel; however, this could not provide any clue about how the structural differences of vascular affect the hydraulic transport in the plant. A mutant tomato (*hws-1*) with wider phloem and narrower xylem rings showed a higher sugar concentration in the phloem exudates and a higher Brix level in the fruit, indicating that the increased phloem area ratio contributes to the higher fruit Brix in tomatoes (Lombardo et al., 2021). In grape, Shigyoku (*Vitis vinifera*) showed 1.8-fold greater phloem area of the pedicel than that of Heukboseok (*V. vinifera*) after veraison, based on the number of cells constituting the phloem, whereas no difference of xylem area was found between them; the cell number and area of the phloem contributed to the accumulation of sugars and the main constituents of the cell wall, thus maintaining the firmness of grapes until late maturity; therefore, the phloem structure was considered to contribute to the increased softening of Heukboseok grapes (Jung et al., 2016). However, our study showed a positive effect of xylem area of the pedicel on the fruit-soluble solids concentration in the fruit; Conference and Nanguoli pears used in our study start to ripe after harvest, a special ripening pattern; therefore, the soluble solids concentration of them came from the degradation of the starch, which depends on the starch accumulation of the stage before fruit maturity. This ripening pattern is different from Akuzuki and Yali, which could reach biological maturity before harvest. The difference in the ripening pattern could explain why our results are different in the mentioned studies on tomato and grape. In a series of studies on cherry, both the phloem and the xylem of the pedicel showed a dynamic flow rate and transpiration rate during the fruit development, which is associated with the fruit enlargement and cracking before and after harvest (Athoo et al., 2015; Brüggenwirth and Knoche, 2015; Knoche et al., 2015; Brüggenwirth et al., 2016). In lemon, the disorganization of vascular tissues of the pedicel with collapsed and deformed xylem disrupted the regular transport of water and nutrients to the growing fruit resulting in fruit cracking (Kaur et al., 2019). An increment of xylem element in the pedicel of kiwifruit during the fruit growth period was found to associate with the higher calcium and magnesium concentration of the fruit, hypothetically through a more efficient translocation of mineral nutrients (Biasi and Altamura, 1996). Studies on the molecular level also found that highly lignified pedicel due to the GA-induced gene regulation (overexpression of *Vv4CL4, VvCCR1L*, and *VvCAD1*) increased grape berry drop (García-Rojas et al., 2018) and that the *LcERF2*-*LcUGE* module regulated the growth of pedicel, concurrent with differential abscission rates and responses to ethylene in litchi (Yi et al., 2021). Together with our findings, the above studies addressed the significant importance of the pedicel in the fruit growth and quality formation in different fruit species, but the underlying physiological and molecular mechanisms need further investigation.

In addition to the microscopic observation, X-ray computed microtomography was also applied in this study, providing a 3D view of the structural properties of the tissues inside the pedicel with reconstructed models (Figures 6, 7). Even though the tissues of pith, fiber, and cortex were rarely discussed in relation to water and nutrients transport, our findings suggested that they likely play a certain role in the fruit growth due to the variations of their characteristics demonstrated in the four pear species (Figure 6); greater surface area ratio of the cortex in Akizuki possibly improved the signal transduction, carbohydrate storage, and the development of the vascular bundles, which contributed to the greater weight of Akizuki fruit; however, this needs further validation. Focusing on the vessels, the porosity, the ratio of the surface area to the volume, and their spatial arrangement showed significant variations across the investigated pear species (Figures 6, 7), indicating the distinction of the hydraulic conductance of the pedicels (further proved in Figure 8). This might determine the transport efficiency and capacity of water and nutrients through the pedicels, differentially contributing to the fruit growth and quality formation across the pear species. In grape, X-ray computed microtomography images provided direct evidence that blockages residing inside the pedicel vessels reduced the hydraulic conductance, and vessel elements were interconnected to keep a partial function during the post veraison (Knipfer et al., 2015). With the help of X-ray computed microtomography, the dynamic formation and spread of the embolism inside the xylem vessels were displayed in living plants (Brodersen et al., 2018; Wason et al., 2021), and they showed significant variations of susceptibility to drought stress among three walnut species (Knipfer et al., 2018). Although the X-ray scanning machine and parameter settings used in the above studies were different from ours, high-resolution observations were all available in the studies, in addition to the isolation of 3D rendering of the single vessel, 3D-reconstructed renderings of the other pedicel tissues; therefore, as long as high-resolution observation is available, X-ray scanning technology is very helpful in plant anatomical studies. Since the lack of living plants used in our experiment, we could not compare the dynamic hydraulic conductance among the pear species. However, we estimated the pedicel hydraulic conductance based on Darcy’s law. The variations in hydraulic conductance of the pedicel emphasized the influence of the pedicel on the fruit growth *via* water and nutrients transport capacity. In similar studies, Darcy’s law was also applied to estimate the pedicel hydraulic conductance in cherry (Brüggenwirth and Knoche, 2015) and flowers (Roddy et al., 2018), suggesting the reliability of the method used in our research.

## Conclusion

As reported widely, X-ray computed microtomography provides plant biologists with a powerful, nondestructive tool to explore the inner workings of plant vascular in incredible detail (McElrone et al., 2013). Our work reconstructed the detailed 3D structure of the pedicel (Figure 6), and the xylem vessel spatial arrangement and the single-vessel isolation were also visualized with remarkable details (Figure 7). Combining the investigation of fruit quality, the statistical analysis of the pedicel structural properties, and the 2D and 3D anatomical observation of the pedicel, we provided direct evidence that pedicel elements contributed distinctively to the fruit characteristics among pear species. These data will help develop a better understanding of the relationship between fruit characteristics and the structural properties of the pedicel. These findings also provide a basis for further anatomical, physiological, and molecular studies into the role of the pedicel in the fruit growth and quality formation.

## Data Availability Statement

The original contributions presented in the study are included in the article/supplementary material, further inquiries can be directed to the corresponding author.

## Author Contributions

ZC analyzed the statistical data and prepared the manuscript. HS conducted the whole experiment. YL and LR took part in the sample collection and the investigation of the fruit characteristics. XX supported the X-ray scanning technique and the helped in data analysis related to 3D reconstruction. DL and RW provided valuable discussions. CM conceived the idea of the research and provided financial support. All authors contributed to the article and approved the submitted version.

## Conflict of Interest

XX is employed by Sanying Precision Instruments. The remaining authors declare that the research was conducted in the absence of any commercial or financial relationships that could be construed as a potential conflict of interest.

## Publisher’s Note

All claims expressed in this article are solely those of the authors and do not necessarily represent those of their affiliated organizations, or those of the publisher, the editors and the reviewers. Any product that may be evaluated in this article, or claim that may be made by its manufacturer, is not guaranteed or endorsed by the publisher.

## References

[B1] Athoo T. O. Winkler A. Knoche M. (2015). Pedicel transpiration in sweet cherry fruit: mechanisms, pathways, and factors. *J. Am. Soc. Hortic. Sci.* 140 136–143.

[B2] Biasi R. Altamura M. M. (1996). Light enhances differentiation of the vascular system in the fruit of actinidia deliciosa. *Physiol. Plant.* 98 28–35.

[B3] Bondada B. Matthews M. Shackel K. (2005). Functional xylem exists in post veraison grape berry. *J. Exp. Bot.* 56 2949–2957. 10.1093/jxb/ern061 16207748

[B4] Brodersen C. R. Knipfer T. McElrone A. J. (2018). *In vivo* visualization of the final stages of xylem vessel refilling in grapevine (*Vitis vinifera*) stems. *New Phytol.* 217 117–126. 10.1111/nph.14811 28940305

[B5] Brüggenwirth M. Knoche M. (2015). Xylem conductance of sweet cherry pedicels. *Trees* 29 1851–1860. 10.1007/s00425-017-2719-3 28623562

[B6] Brüggenwirth M. Winkler A. Knoche M. (2016). Xylem, phloem, and transpiration flows in developing sweet cherry fruit. *Trees* 30 1–10. 10.1016/j.jplph.2019.04.007 31022664

[B7] Bussières P. (2002). Water import in the young tomato fruit limited by pedicel resistance and calyx transpiration. *Funct. Plant Biol.* 29 631–641. 10.1071/PP00144 32689508

[B8] Bustan A. Erner Y. Goldschmidt E. (1995). Interactions between developing Citrus fruits and their supportive vascular system. *Ann. Bot.* 76 657–666.

[B9] Choat B. Gambetta G. A. Shackel K. A. Matthews M. A. (2009). Vascular function in grape berries across development and its relevance to apparent hydraulic isolation. *Plant Physiol.* 151 1677–1687. 10.1104/pp.109.143172 19741048 PMC2773088

[B10] Cui Z. Jiao Q. Wang R. Ma C. (2020). Investigation and analysis of relationship between mineral elements alteration and cork spot physiological disorder of Chinese pear ‘Chili’ (*Pyrus bretschneideri* Rehd.). *Sci. Hortic.* 260:108883.

[B11] de Oliveira R. H. Milanez C. Moraes-Dallaqua M. A. Rosolem C. A. (2006). Boron deficiency inhibits petiole and peduncle cell development and reduces growth of cotton. *J. Plant Nutr.* 29 2035–2048.

[B12] Drazeta L. Lang A. Cappellini C. Hall A. J. Jameson P. E. (2004). Vessel differentiation in the pedicel of apple and the effects of auxin transport inhibition. *Physiol. Plant* 120 162–170. 10.1111/j.0031-9317.2004.0220.x 15032888

[B13] Feldkamp L. A. Davis L. C. Kress J. W. (1984). Practical cone-beam algorithm. *J. Opt. Soc. Am. A* 1 612–619.

[B14] García-Rojas M. Meneses M. Oviedo K. Carrasco C. Defilippi B. González-Agüero M. (2018). Exogenous gibberellic acid application induces the overexpression of key genes for pedicel lignification and an increase in berry drop in table grape. *Plant Physiol. Biochem.* 126 32–38. 10.1016/j.plaphy.2018.02.009 29499433

[B15] Gilliham M. Dayod M. Hocking B. J. Xu B. Conn S. J. Kaiser B. N. (2011). Calcium delivery and storage in plant leaves: exploring the link with water flow. *J. Exp. Bot.* 62 2233–2250. 10.1093/jxb/err111 21511913

[B16] Greenspan M. D. Schultz H. R. Matthews M. A. (1996). Field evaluation of water transport in grape berries during water deficits. *Physiol. Plant* 97 55–62. 10.1071/FP02115 32689050

[B17] Greenspan M. D. Shackel K. A. Matthews M. A. (1994). Developmental changes in the diurnal water budget of the grape berry exposed to water deficits. *Plant Cell Environ.* 17 811–820.

[B18] Horbens M. Branke D. Gärtner R. Voigt A. Stenger F. Neinhuis C. (2015). Multi-scale simulation of plant stem reinforcement by brachysclereids: a case study in apple fruit peduncles. *J. Struct. Biol.* 192 116–126. 10.1016/j.jsb.2015.08.002 26278981

[B19] Jung M. Oh J. P. Kim J. Park Y. Park H. S. (2016). Differences in softening of ‘Shigyoku’ and ‘Heukboseok’ grapes during harvest period appears to be related to differences of pedicel vascular bundle. *Korean J. Hortic. Sci. Technol.* 34 692–700.

[B20] Kaur R. Kaur N. Singh H. (2019). Pericarp and pedicel anatomy in relation to fruit cracking in lemon (*Citrus* limon l burm.). *Sci. Hortic.* 246 462–468.

[B21] Kawamoto T. (2003). Use of a new adhesive film for the preparation of multi-purpose fresh-frozen sections from hard tissues, whole-animals, insects and plants. *Arch. Histol. Cytol.* 66 123–143. 10.1679/aohc.66.123 12846553

[B22] Knipfer T. Barrios-Masias F. H. Cuneo I. F. Bouda M. Mcelrone A. J. (2018). Variations in xylem embolism susceptibility under drought between intact saplings of three walnut species. *Tree Physiol.* 38, 1–13. 10.1093/treephys/tpy049 29850910

[B23] Knipfer T. Fei J. Gambetta G. A. Mcelrone A. J. Matthews M. A. (2015). Water transport properties of the grape pedicel during fruit development: insights into xylem anatomy and function using microtomography. *Plant Physiol.* 168 1590–1602. 10.1104/pp.15.00031 26077763 PMC4528730

[B24] Knoche M. Athoo T. O. Winkler A. Brüggenwirth M. (2015). Postharvest osmotic dehydration of pedicels of sweet cherry fruit. *Postharvest Biol. Technol.* 108 86–90.

[B25] Lee D. R. (1989). Vasculature of the abscission zone of tomato fruit: implications for transport. *Can. J. Bot.* 67 1898–1902.

[B26] Li H. Zhang X. Hou X. Du T. (2021). Developmental and water deficit-induced changes in hydraulic properties and xylem anatomy of tomato fruit and pedicel. *J. Exp. Bot.* 72 2741–2756. 10.1093/jxb/erab001 33420789

[B27] Lombardo F. Gramazio P. Ezura H. (2021). Increase in phloem area in the tomato hawaiian skirt mutant is associated with enhanced sugar transport. *Genes* 12:932. 10.3390/genes12060932 34207298 PMC8234570

[B28] McElrone A. J. Choat B. Dilworth Y. P. Macdowell A. A. Brodersen C. R. (2013). Using high resolution computed tomography to visualize the three-dimensional structure and function of plant vasculature. *J. Vis. Exp.* 74:50162. 10.3791/50162 23609036 PMC3643333

[B29] Nii N. (1980). Seasonal changes in growth and enlargement of the japanese pear fruit, pyrus serotina cv shinsheiki, in relation to vascular bundle development in the pedicel and flesh. *J. Hort.* 55 385–396.

[B30] Nii N. (1998). *Fruit Growth And Development.* Tokyo: Asakura Press.

[B31] Park Y. Park H. S. (2017). Microstructural changes in the fruit and pedicel of ‘wonhwang’ oriental pear induced by exogenous gibberellins. *Sci. Hortic.* 222 1–6.

[B32] Rančić D. Quarrie S. P. Radosevic R. Terzic M. Pecinar I. Stikic R. (2010). The application of various anatomical techniques for studying the hydraulic network in tomato fruit pedicels. *Protoplasma* 246 25–31. 10.1007/s00709-010-0115-y 20165892

[B33] Rančić D. Quarrie S. P. Terzic M. Savic S. Stikic R. (2008). Comparison of light and fluorescence microscopy for xylem analysis in tomato pedicels during fruit development. *J. Microsc.* 232 618–622. 10.1111/j.1365-2818.2008.02127.x 19094049

[B34] Roddy A. Simonin K. McCulloh K. Brodersen C. Dawson T. (2018). Water relations of Calycanthus flowers: hydraulic conductance, capacitance, and embolism resistance. *Plant Cell Environ.* 41 2250–2262. 29603273 10.1111/pce.13205

[B35] Setiamihardja R. Knavel D. E. (1990). Association of pedicel length and diameter with fruit length and diameter and ease of fruit detachment in pepper. *J. Am. Soc. Hortic. Sci.* 115 677–681.

[B36] Song W. Yi J. Kurniadinata O. F. Wang H. Huang X. (2018). Linking fruit ca uptake capacity to fruit growth and pedicel anatomy, a cross-species study. *Front. Plant Sci.* 9:575. 10.3389/fpls.2018.00575 29868049 PMC5954447

[B37] Song W. P. Chen W. Yi J. W. Wang H. C. Huang X. M. (2018). Ca distribution pattern in litchi fruit and pedicel and impact of Ca channel inhibitor, La3. *Front. Plant Sci.* 8:2228. 10.3389/fpls.2017.02228 29375603 PMC5767242

[B38] Van Ieperen W. Volkov V. S. Van Meeteren U. (2003). Distribution of xylem hydraulic resistance in fruiting truss of tomato influenced by water stress. *J. Exp. Bot.* 54 317–324. 10.1093/jxb/erg010 12493859

[B39] Wason J. Bouda M. Lee E. F. Mcelrone A. J. Brodersen C. (2021). Xylem network connectivity and embolism spread in grapevine (*Vitis vinifera* l.). *Plant Physiol.* 186 373–387. 10.1093/plphys/kiab045 33576825 PMC8154096

[B40] Yi J. W. Wang Y. Ma X. S. Zhang J. Q. Zhao M. L. Huang X. M. (2021). LcERF2 modulates cell wall metabolism by directly targeting a UDP-glucose-4-epimerase gene to regulate pedicel development and fruit abscission of litchi. *Plant J.* 106 801–816. 10.1111/tpj.15201 33595139

[B41] Zhang C. Kenji T. Fumio T. Kazuhiro M. Akira Y. (2005). 13c-photosynthate accumulation in Japanese pear fruit during the period of rapid fruit growth is limited by the sink strength of fruit rather than by the transport capacity of the pedicel. *J. Exp. Bot.* 56 2713–2719. 10.1093/jxb/eri264 16131508

